# Brain outcomes with lifestyle change in adults with Down syndrome: Rationale and design for a 12‐month randomized trial

**DOI:** 10.1002/alz.70219

**Published:** 2025-05-06

**Authors:** Lauren T. Ptomey, Joseph E. Donnelly, Jeffery Burns, Jill Morris, Sigan Hartley, Laura E. Martin, In‐Young Choi, Phill Lee, Yanming Li, Suzanne Hunt, Joseph R. Sherman, Jessica C. Danon, Morgan G. Brucks, Lyndsie Koon, Kendra Spaeth, Debra K. Sullivan

**Affiliations:** ^1^ Department of Internal Medicine The University of Kansas Medical Center Kansas City Kansas USA; ^2^ Department of Neurology The University of Kansas Medical Center Kansas City Kansas USA; ^3^ Waisman Center University of Wisconsin Madison Madison Wisconsin USA; ^4^ Department of Population Health University of Kansas Medical Center Kansas City Kansas USA; ^5^ Hoglund Biomedical Imaging Center University of Kansas Medical Center Kansas City Kansas USA; ^6^ Department of Radiology University of Kansas Medical Center Kansas City Kansas USA; ^7^ Department of Biostatistics & Data Science University of Kansas Medical Center Kansas City Kansas USA; ^8^ Life Span Institute University of Kansas Lawerence Kansas USA; ^9^ Department of Nutrition University of Kansas Medical Center Kansas City Kansas USA

**Keywords:** Alzheimer's disease, biomarkers, cognition, diet, Down syndrome, MIND, nutrition, technology, weight loss

## Abstract

Most adults with Down syndrome (DS) will develop pathology associated with Alzheimer's disease (AD). By age 65, the cumulative incidence of dementia exceeds 90%. The available data from trials in typically developed adults suggest the potential for intentional weight loss and changes in dietary intake to prevent or delay the development of AD. However, the potential of weight loss to prevent or delay AD in adults with DS has not been previously examined. In this article, we describe the methods and rationale for a randomized trial that will evaluate the impact of weight loss and dietary intake on factors that may delay the development of AD in adults with DS including cognition, plasma biomarkers, and brain structure. The results of this trial will provide information on if non‐pharmacological approaches could delay onset of AD in adults with DS.

**Clinical Trials Registration**: NCT05985486

**Highlights:**

- By the age of 65 the cumulative incidence of dementia exceeds 90% in adults with DS.
- There is a paucity of non‐pharmacological interventions to delay AD in adults with DS.
- Clinical trials in adults without DS suggest that intentional weight loss may improve cognitive function and reduce the risk of developing AD.
- A randomized trial exploring the impact of weight loss on AD biomarkers in adults with DS is currently underway.
- This trial will explore if non‐pharmacological approaches could delay onset of AD in adults with DS.

## 1 INTRODUCTION

Down syndrome (DS), or trisomy 21, is the most common chromosomal abnormality associated with an intellectual disability (ID).^1^ Most adults with DS will develop pathology related with Alzheimer's disease (AD).^2^ The median age of dementia onset is 53.8 years,^3^ and the lifetime incidence is 90%.^2^ High rates of AD in DS are the result of the overproduction of amyloid‐beta (Aβ) from the additional copy of the amyloid precursor protein found on chromosome 21. Aβ accelerates neurodegeneration, oxidative stress, plaque deposition, and early development of AD‐like pathology in individuals with DS.^4^ Research suggests that deposition of Aβ occurs decades earlier in adults with DS compared with typically developed adults, with a mean age of AD onset of 53.8 years. However, a recent review found significant individual variability in onset (25 years),^5^ suggesting that the risk and timing of dementia is modifiable. Lifestyle factors are posited to explain part of this variability. In adults without DS, lifestyle factors contribute to 45% of late onset AD.^6^

Evidence from both epidemiological and clinical observations suggest that in the general population, mid‐life obesity is a significant risk factor for later‐life dementia (including AD) independent of comorbidities such as hypertension and diabetes.^6^ However, the impact of obesity on AD risk in adults with DS remains largely unexplored. To our knowledge, only one study has examined this relationship, finding an association between body mass index (BMI) and Free and Cued Recall in 66 adults with DS without dementia.^7^ Given the high prevalence of obesity in adults with DS (∼81%)^8^ further research is needed to better understand its role in AD risk and progression in this population.

Similarly, the available data from trials in typically developed adults suggest the potential for weight loss achieved through a reduced energy diet and/or consuming a low glycemic load, low saturated fat, high fruit and vegetable diet to prevent or delay the development of AD.^9^, ^10^, ^11^, ^12^ Additionally, in adults without DS, the Mediterranean‐DASH diet Intervention for Neurodegenerative Delay (MIND) dietary pattern is associated with improved cognitive function and reduced incidence of AD.^13^, ^14^, ^15^ However, the potential of weight loss and dietary interventions to prevent or delay AD in mid‐life adults with DS has not been previously examined.

Our group has developed a multi‐component intervention, including a reduced energy‐enhanced Stop Light Diet (eSLD), individual behavioral counseling/education, self‐monitoring, and promotion of physical activity, which has been shown to achieve and sustain clinically relevant weight loss (≥5%) and improvements in diet quality in adults with ID. The eSLD includes daily consumption of two portion‐controlled entrées (∼200 kcal each), two low‐calorie/high protein shakes (∼100 kcal each), a minimum of five servings of fruits/vegetables, and ad libitum noncaloric beverages. Participants are asked to select additional low energy food, if desired, using the SLD system: green (low energy), yellow (moderate energy), and red (high energy). Results from three completed trials which have included a total of 335 adults with ID including DS, have demonstrated improved diet quality and clinically relevant weight loss at both 6 months (−6.2) and 12 months (−6.8%) using the eSLD.^16^, ^17^, ^18^ This article provides the methods and rationale for a randomized trial that will use the eSLD to evaluate the impact of weight loss and dietary intake on factors that may prevent or delay the development of AD in adults with DS.

## 2 METHODS

### 2.1 Overview of study design

This is a 12‐month randomized trial in 81 non‐demented adults with DS. Participants will be randomized (2:1) to either a weight loss or a general health education control group. The weight loss arm will follow the protocol used in our previous trials,^16^, ^17^, ^18^ plus recommendations from the MIND diet,^13^, ^14^ to increase antioxidant consumption and further improve diet quality. Participants and a study partner will be asked to attend monthly behavioral counseling/education sessions with a trained interventionist delivered remotely using Zoom video conferencing (San Jose, CA, USA) and self‐monitor diet and body weight using commercially available Web‐based applications. Participants and a study partner in the control arm will be asked to attend monthly behavioral counseling/education sessions with a trained interventionist delivered remotely and will receive education on general health education topics such as injury prevention, oral health, sleep, hydration, exercise and physical activity, and healthy eating. Outcomes assessed across 12 months will include (1) biomarkers related to AD including plasma amyloid beta 42:40 ratio (Aβ 42/40), phosphorylated Tau (pTau217), neurofilament light (NfL), and glial fibrillary acidic protein (GFAP); (2) cognitive function using both directly‐administered and informant‐based assessments; (3) cardiometabolic biomarkers such as lipid levels, blood glucose levels, and inflammatory markers; (4) cerebral antioxidants and brain volume assessed by magnetic resonance spectroscopy; and (5) dietary intake assessed using 3‐day photo‐assisted food record and skin carotenoid content using a Veggie Meter. Our analysis will include three aims (Table 1). This trial will provide preliminary data on the impact of weight loss and improved diet quality on cognitive function and factors associated with the development of AD.

**TABLE 1:** Study aims for a 12‐month weight loss intervention in adults with Down syndrome.

Aim 1	Calculate effect sizes for changes in plasma biomarkers related to AD, cardiometabolic biomarkers, and cognitive function in both intervention arms across 12 months.
Aim 2	Calculate effect sizes for changes in cerebral antioxidants and brain volume in a subsample of participants in both intervention arms across 12 months.
Aim 3	Explore the independent effects of weight loss or diet quality on changes in plasma biomarkers related to AD, cerebral antioxidants and brain volume, and cognitive function across 12 months.

Abbreviation: AD, Alzheimer's disease.

### 2.2 Participant eligibility

To enhance generalizability, individuals with DS on medications for common chronic diseases (i.e., depression, hypertension, type 2 diabetes), using dietary supplements, or consuming a gluten‐free diet (i.e., celiac disease) will not be excluded. Random assignment should distribute these characteristics equally across interventions arms. Additionally, we will control for baseline characteristics in our analysis. Inclusion and exclusion criteria are provided in Table 2.

**TABLE 2:** Participant eligibility criteria for a 12‐month weight loss intervention in adults with Down syndrome.

Inclusion	
Residential status	Living at home with a parent/guardian or in a supported living environment with a family member, caregiver, or direct support professional who agrees to serve as a study partner for the entire 12‐month intervention
Age	18–64 years
Diagnosis	Down syndrome
BMI	25–50 kg/m^2^
Communication	Sufficient functional ability to understand directions, communicate preferences, wants, and needs through spoken language
Exclusion	
Dementia	Diagnosis of dementia as determined by the Dementia Screening Questionnaire for Individuals with Intellectual Disabilities (DSQIID)
Health concerns	Serious medical risk (e.g., recent cancer, recent heart attack, stroke, pregnancy, angioplasty) as determined by the health history form Insulin‐dependent diabetes Dairy allergy Swallowing disorders that require a thickened liquid diet
Medications	Individuals on GLP‐1 antagonist Individuals taking warfarin or other blood thinner medications Individuals taking anti‐amyloid medications or other medications related to the treatment of Alzheimer's disease
Other	Participation in a weight management program involving diet and PA in the past 6 months Unwilling to be randomized

Abbreviations: BMI, body mass index; GLP‐1, glucagon‐like peptide‐1; PA, physical activity.

### 2.3 Recruitment/Randomization

Project staff will contact local community agencies serving adults with DS, case managers, and Community Developmental Disabilities Organizations by mail/email, provide presentations at community agencies and local events, and distribute newsletters describing the project. Interested parents/guardians, caregivers, or adults with DS will be asked to contact the study coordinator via email, our website, or a dedicated toll‐free study phone number that will be included in all recruitment materials. The study coordinator will contact the interested individuals by phone to answer questions and conduct an initial eligibility screen. Video conference visits will be scheduled with those remaining interested, and potentially eligible, to obtain informed consent (participants or legal guardian) and participant assent (if not their own legal guardian) and determine final eligibility based on the DSQIID scores. Participants found to be ineligible will be provided with written materials describing available resources for weight loss. Participants will be stratified by age (18–30 vs. > 30 years) and sequentially computer‐randomized by the study statistician with 2:1 allocation to the weight loss or control arms. This randomization scheme was selected to include more participants in the weight loss arm which will allow for a larger sample size to assess the association of weight loss and changes in dietary intake with changes in biomarkers and cognitive function. Intervention assignments will be concealed and delivered to the study coordinator prior to participant orientation sessions described below.

### 2.4 Study partner role

Adults with DS receive assistance from family members and direct support professionals with decisions regarding meal planning, food shopping, and meal preparation, thus, playing an important role in the success of weight loss interventions in this population. For this trial, we will recruit either a parent/guardian, other family member, or direct support professional for those living in group homes to serve as a study partner for each participant. Study partners for participants in both intervention arms will be asked to attend the orientation and all monthly meetings with the interventionist and to provide transportation to our facilities for all outcome assessments. Study partners for participants in the weight loss arm will be asked to support, encourage, and assist participants in complying with the study protocol including the selection and preparation of foods consistent with the dietary recommendations, and assisting participants with self‐monitoring of both dietary intake and body weight.

### 2.5 Intervention components

#### 2.5.1 Weight loss arm

##### Orientation

Interventionists will conduct two ∼60 min home visits with participants and study partners prior to initiating the intervention. Interventionists will include detailed descriptions of all components of the weight loss intervention protocol. Participants will be provided with an iPad (Apple Inc., Cupertino, CA, USA). All iPads will be pre‐loaded with video conferencing (Zoom) and diet and weight tracking software (CoachCare platform). Participants will receive a tutorial on the use of the iPad, video conferencing and diet tracking software, and wireless scales apps/devices used for self‐monitoring diet and weight.

##### Diet

Reduced energy intake (goal: 1200–1800 kcal/day) will be achieved using an eSLD. Participants will be asked to consume a minimum daily total of two entrées (∼200–300 kcal each, saturated fat ≤ 3 g), one high‐protein shake (∼100 kcal each), five servings of fruits/vegetables, and ad libitum non‐caloric beverages. To increase antioxidant content and improve diet quality, we will incorporate specific recommendations from the MIND diet. Participants and study partners will be trained to use a color‐coded chart that we have developed which categorizes foods based on the Stop Light system to assist in meal planning, grocery shopping, decisions regarding snack foods, and compliance with the diet in special situations such as eating away from home (restaurants, parties, etc.). Participants will be asked to purchase and prepare portion‐controlled entrées from a list meeting the caloric/fat requirements, as well as all fruits and vegetables, and non‐caloric beverages. The study will provide one low‐calorie/high protein, dairy‐based shake per day which will be shipped to the participant's residence monthly.

##### Physical activity

Moderate‐to‐vigorous physical activity (MVPA [≥ 3 METs]) is recommended as part of multi‐component weight management interventions^19^ and also has numerous additional health benefits for adults with DS which include maintaining components of cognition, including attention, memory, and executive function.^7^, ^20^ Thus, promoting recommended levels of MVPA in the weight management intervention may potentially confound our results. However, given the potential health benefits of MVPA, we felt that it would be unethical to recommend that participants refrain from engaging in MVPA. We chose to recommend 150 min per week of MVPA following the Physical Activity Guidelines for Americans.^21^ We will not ask participants to set MVPA goals during interventionist meetings or to self‐monitor MVPA across the intervention for the proposed trial. Data from our previous trials in adults with ID which used the same 150 min per week MVPA recommendation in addition to participant MVPA goal setting during meetings with interventionists and continuous self‐monitored MVPA using a Fitbit were generally unsuccessful in increasing MVPA.^17^ Thus, we anticipate minimal increases in MVPA across the intervention in the weight loss arm. However, to control for any between‐arm differences in MVPA across the intervention, we will assess MVPA using portable accelerometers (see the Outcomes section) in both the weight loss and control arms at baseline 6 and 12 months.

##### Self‐monitoring

Participants will be asked but not required to monitor diet daily and body weight monthly over the 12‐month intervention. For this trial, participants with DS with assistance from their study partner will log food/beverages (meals/snacks) using the CoachCare platform by entering the food name and selecting the portion size, or by scanning the bar code of the food item using the iPad. A bar graph displays calories consumed compared with the goal, providing immediate feedback. Reminders will be sent via the app if no information is reported for a given meal. Participants will self‐weigh during monthly education/behavioral counseling video sessions using the wireless digital scale provided by the study which automatically syncs with the CoachCare app and updates a visual display of weight change. CoachCare platform data, which will be accessible in real time to interventionists, will be used *only* to provide motivation and feedback during the education/behavioral counseling sessions.

##### Education/behavioral counseling sessions

These sessions (∼45 min) will be conducted via a Health Insurance Portability and Accountability Act (HIPPA) compliant video conference meeting over Zoom with a trained interventionist, monthly across the 12‐month intervention. Education/behavioral counseling sessions will include a review of participants self‐monitored diet and weight data, individual problem‐solving and goal setting, and reviewing an interactive lesson on a weight management topic such as energy density (increase volume, not calories), the importance of fruits/vegetables, eating in social situations, basic cooking skills, and so forth. Participants and study partners will receive an automated text message and email bi‐weekly using the CoachCare app encouraging them to apply the topics discussed in the monthly lesson.

#### 2.5.2 Control arm: General health education

##### Orientation

Similar to the weight loss arm, interventionists will conduct a 30 min visit with participants and study partners prior to initiating the intervention to provide an overall description of the health education curriculum and the meeting schedule. Participants will be provided with and receive a tutorial on the use of an iPad loaded with Zoom video conferencing software.

##### Education sessions

Education sessions (∼30 min) will be conducted via a HIPPA compliant Zoom meeting, monthly across the 12‐month intervention. Topics include injury prevention, oral health, sleep hygiene, hydration, exercise and physical activity, healthy eating, importance of health behaviors for disease prevention, and so forth. Sessions will provide information regarding these topics and will not ask participants to self‐monitor behaviors or make lifestyle changes relative to any of these topics. Participants and study partners will receive an automated text message and email bi‐weekly reviewing the previous month's topic.

### 2.6 Intervention fidelity

Twenty percent of education sessions in both the weight loss and control arms will be video recorded and reviewed by study staff. The content delivered during each session will be compared with a checklist of scheduled content. Interventionists presenting < 80% of the scheduled content for any session will receive additional training and will be dismissed if the problem recurs.

### 2.7 Outcome assessments

All assessments will be completed by appropriately trained staff blinded to the intervention arm (Table 3). All outcomes will be assessed during two separate visits to an academic medical center. Visit 1 will include assessments of height, weight and waist circumference, blood pressure, a blood draw for plasma biomarkers, cognitive function, diet intake, and skin carotenoid content. These assessments will require ∼ 120 min to complete. Visit 2, which is optional, will include neuroimaging and will require ∼ 60 min to complete. Staff will receive refresher training and complete reliability assessments for all physical measures two to three times per year.

**TABLE 3:** Outcome assessments across a 12‐month weight loss intervention in adults with Down syndrome.

Assessments	Pre‐study	Baseline	6 months	12 months
*Screening*				
DSQIDD	X			
Demographic health questionnaire	X			
Participant health history	X			
*Visit 1*				
Anthropometrics and blood pressure		X	X	X
Blood draw		X	X	X
3‐Day photo assisted food record		X	X	X
Skin carotenoid content		X	X	X
Kaufman Brief Intelligence Test (KBIT‐2)		X		
Modified Cued Recall task		X	X	X
Down Syndrome Mental Status Examination		X	X	X
Stroop dog and cat		X	X	X
NTG‐Early Detection Screen for Dementia (NTG‐EDSD)		X	X	X
Dementia questionnaire for people with learning disabilities (DLD)		X	X	X
Neuropsychiatric Inventory (NPI)		X	X	X
Vineland Adaptive Behavior Scales		X	X	X
Accelerometer		X	X	X
*Visit 2*				
MRI/MRS		X		X

Abbreviations: DSQIID, Dementia Screening Questionnaire for Individuals with Intellectual Disabilities; MRI, magnetic resonance imaging; MRS, magnetic resonance spectroscopy; NTG, National Task Group.

#### 2.7.1 Visit 1: Clinical and cognitive assessment

##### Blood draw

We will collect a blood sample to analyze plasma biomarkers related to AD (Aβ42/40, pTau217, NfL, and GFAP) and cardiometabolic biomarkers (lipids, glucose, glycated hemoglobin [HbA1c], inflammatory markers). Phlebotomy will occur after an overnight fast (>8 h). AD biomarkers will be collected using ethylenediaminetetraacetic acid (EDTA) as an anticoagulant, inverted 8× to mix and processed at 1800 × *g* for 10 min at 4°C prior to storage at −80°C. Cardiometabolic biomarkers will be collected into gold top serum separator tubes, inverted 8× to mix, and allowed to clot for 30 min at room temperature prior to centrifugation at 1500 × *g* for 10 min prior to analysis. AD biomarker analyses will be performed on the first freeze‐thaw cycle using the Simoa HD‐X (Quanterix, Billerica, MA, USA). Cardiometabolic biomarkers will be analyzed in a clinical laboratory.

##### Anthropometrics

A clinician will measure weight, height, and waist circumference. Weight will be measured on a calibrated scale to the nearest 0.1 kg. Standing height will be measured with a stadiometer. BMI will be calculated as weight (kg)/height (m^2^). Waist circumference will be assessed using standard procedures such as those described by Lohman et al.^22^ Systolic and diastolic blood pressure will be assessed with two measurements using a calibrated automatic cuff on the left arm with the participant in a seated position.

##### Dietary intake

Dietary intake will be assessed using photo‐assisted food records^23^ over 3 consecutive days (2 week days/1 weekend day) starting the week prior to scheduled outcome assessments. Participants will be encouraged to take before and after photographs of all foods and beverages consumed using the iPad camera which automatically date and time stamps photos for easy identification. Hard copy records will be completed to account for details not discernible from photos (e.g., skim or whole milk). During outcome visits, staff will review photos and food records and clarify ambiguities. If no photo is available for a meal, food record data will be used.

##### Skin carotenoid content

Skin carotenoid content will be assessed by a finger scan using the Veggie Meter (Longevity Link Corporation), as a biomarker of fruit and vegetable intake.^24^

##### Cognitive assessment

A trained psychometrist will administer a psychometric battery to assess broad cognitive abilities using the Down Syndrome Mental Status Exam (DSMSE),^25^ modified Cued Recall Task,^26^ and The Modified Cats and Dogs Task.^27^ Additionally at baseline, we will collect the Kaufman Brief Intelligence Test Second Edition^28^ to measure premorbid ID level. These cognitive measures have been shown to be sensitive to early AD‐related cognitive decline^26^, ^29^ and are being used in national cohorts examining AD in DS^30^ which will allow for harmonization of data.

##### Study partner outcomes

Study partner‐based interviews will focus on participants’ adaptive, cognitive, neuropsychiatric status and possible observed AD symptomatology and will include the Dementia Questionnaire for People with Learning Disabilities (DLD),^31^ the National Task Group Early Detection Screen for Dementia (NTG‐EDSD),^25^ The Vineland Adaptive Behavior Scales, third edition (VABS‐3),^32^ and The Neuropsychiatric Inventory (NPI‐Q).^33^ These informant‐based cognitive measures are associated with early changes in plasma and imaging assessed biomarkers related to AD.^29^, ^34^, ^35^

##### Physical activity

Participants will be asked to wear an ActiGraph (ActiGraph GT9X, ArchiMed Co, Lyon, Auvergne‐Rhone‐Alpes, France) tri‐axial accelerometer (3.3 cm × 4.6 cm × 3.5 cm, wt. = 19 g., dynamic range ± 8 g) watch on the non‐dominant wrist for 7 consecutive days, including at night. ActiGraphs will be administered at the study visit and returned by postage paid mail following completion of all assessments.

#### 2.7.2 Visit 2: Neuroimaging (optional)

This visit will last approximately 1 h and occur at baseline and 12 months. Scanning will be performed on a 3T Siemens Skyra scanner using a 20‐channel head coil. A T1‐weighted structural scan will be completed using 3D multi echo MPRAGE (MEMPRAGE) with real‐time motion correction and selective reacquisition^36^ (repetition time/inversion time/echo time [TR/TI/TE] = 2500/1300/1.79,3.65,5,51,7.37 ms, flip angle = 7°, field of view [FOV] = 256, matrix = 256 × 256 × 176, resolution = 1 mm^3^, scan time = 6.5 min). Volumetric analysis will be performed using segmentation and brain region parcellation pipelines implemented in FreeSurfer.^37^, ^38^ Regions of interest will include white matter, total gray matter, total intercranial volume, and hippocampal volume. To explore how the diet intervention impacts nutrient concentrations in the brain, brain glutathione (GSH)^39^ and vitamin C^40^ scans will be performed in an axial slice covering frontal and parietal regions using the doubly‐selective multiple quantum (MQ) CSI sequences with real‐time motion/shim correction (TE/TR = 115/1750 ms for GSH, TE/TR = 124/1750 ms for vitamin C, FOV = 20 cm, matrix = 10 × 10, slice thickness = 2.5 cm, scan time = 11.5 min each). Previous research in cognitively normal older adults suggest dietary interventions can lead to significant changes in GSH levels in as little as 12 weeks.^41^ Brain antioxidant signals will be quantified using the internal concentration reference method utilizing unsuppressed brain water signals. We will use previously established quantification procedures using in‐house data processing program written in Interactive Data Language (IDL; L3Harris, Bloomfield, CO, USA).^39^ Brain GSH and vitamin C concentrations in each voxel will be corrected for partial volume effect due to brain atrophy using effective tissue volume fraction calculated from magnetization‐prepared rapid gradient echo (MPRAGE) magnetic resonance imaging (MRI).^42^ We will use all the voxels that pass the preset criteria for spectral quality and tissue volume fraction in both region‐of‐interest analysis^39^ and voxel‐based analysis. We will also determine antioxidant concentrations in gray and white matter using well‐established regression‐based methods^42^ to examine potential differences of antioxidant concentrations in gray and white matter, and differential changes in gray matter and white matter after intervention.

#### 2.7.3 Exit interview

At the completion of the 12‐month intervention, we will conduct structured interviews by Zoom with a 20% random sample of participants and study partners from each intervention arm to gather information that might be useful for improving the intervention. Qualitative content analysis (ATLAS.ti 6.2) will be conducted to search for broad themes across interviews.

#### 2.7.4 Process measures

Attendance at education/behavioral counseling sessions will be obtained from interventionist records and expressed as the percentage of possible sessions attended, and compliance with self‐monitoring of diet (weight loss arm only) will be assessed as the percentage of prescribed monitoring days completed across 12 months.

### 2.8 Incentives

Participants will receive $5.00 for each monthly session attended (up to $60 total) and will be allowed to keep the iPad and wireless scale (weight loss group only) upon study completion. Additionally, to compensate for time and travel, participants will receive $100 for completion of the outcome assessments at baseline, 6 and 12 months ($300 total for all three testing time points).

### 2.9 Analysis/sample size

#### 2.9.1 Sample size

The primary aim is to estimate the effect of a 12‐month weight loss intervention relative to control to determine if the intervention impacts relevant outcome variables, and if so, to precisely quantify the observed effect. The sample size was estimated using simulation with regard to plasma NfL based on the observation that changes in this biomarker is an early marker of AD in adults with DS.^2^ A recent 2021 paper reported an annual change in NfL of 3% in asymptomatic non‐progressive AD patients.^43^ Thus, we simulated annual data at baseline, 6 and 12 months assuming an average 3% annual increase in NfL for all participants in the control arm and an average change of 1.35% in the intervention arm which corresponds to a “small” Cohen's h effect size of 0.12. We modeled the between arm difference in NfL using a linear mixed model with a random intercept for each participant; with a sample size of 81 (*n* = 54 intervention; *n* = 27 control) and an estimated attrition rate of 20%. With these assumptions, we will be able to estimate the precision of the difference in the annualized increase in NfL with a 95% confidence interval of width 3%. Similar mixed models will be fit for all seven outcome variable categories to estimate the effects across all the domains of interest. A major concern with using estimated effects from pilot studies to power larger trials is that they are highly variable. This sample size and approach will guarantee good precision for our estimates which is especially important considering that there are currently no data regarding clinically relevant changes to use for powering a larger trial.

#### 2.9.2 Analysis

We will assess plasma biomarkers related to AD, cardiometabolic biomarkers, cognitive function, energy and macronutrient intake, and diet quality at baseline, 6 and 12 months. We will assess cerebral antioxidants and brain volume at baseline and 12 months. We will calculate the mean, standard deviation, and 95% confidence intervals in both the intervention and control arms for each of these outcome variables at each time point, and for the change from baseline to each time point. This descriptive information will be summarized graphically which will aid in determining if, and when, differences in outcome variables arise among and across the two arms. Linear mixed models with an autoregressive correlation structure and a random intercept for each participant will be used to model the longitudinal serial correlation and baseline between‐subject variation. The model will include a main effect for intervention arm, and time and an arm‐by‐time interaction. The point estimate and 95% confidence interval for the arm‐by‐time interaction evaluated at both 6 and 12 months will be used to estimate the between arm effect size. Mixed models will be adjusted for age, sex, ethnicity/race, MVPA, premorbid ID, number of study participants living in the same household, intervention attendance, and intervention arm. Model fit will be assessed using diagnostic plots and comparing likelihood values between alternative model formulations or transformations, when appropriate. No multiplicity adjustment will be conducted since no hypotheses will be tested.

To determine the independent effects of weight loss or diet quality on changes in plasma biomarkers related to AD (Aβ 42/40, pTau217, and NfL), cerebral antioxidants, brain volume, cardiometabolic biomarkers, and cognitive function we will conduct a set of nested likelihood ratio tests (LRT). The goal of this analysis is to determine if weight loss, diet quality or both should be targeted in future trials for the prevention of AD in adults with DS. No adjustment for multiple testing will be conducted because rejection of any of the nested comparisons will imply that a change in a greater number of underlying covariates, for example, weight loss and diet compared to weight loss alone are needed to elicit changes in biomarkers.

## 3 DISCUSSION

Most adults with DS will develop pathology related with AD.^2^, ^4^ While therapeutic strategies to prevent or treat AD exist for the general population, similar strategies for adults with DS are still in the early stages of development.^44^ Although clinical trials have investigated various drugs and supplements, such as donepezil,^45^ rivastigmine,^46^ memantine,^47^ and vitamin E,^48^ none have been proven to be both safe and effective for preventing AD in adults with DS. Currently, there are new pharmacological clinical trials underway. However, these trials are for secondary prevention or treatment in adults with DS who already have Aβ positivity and aim to reduce or prevent Aβ accumulation. Additionally, most of these trials have a chance of moderate to serious adverse events, such as amyloid‐related imaging abnormalities (ARIA), which can manifest as brain swelling or brain bleeding.^49^ Thus, non‐pharmacological lifestyle trials which focus on primary and secondary prevention of AD may be a safer alternative for early prevention, or even a strategy to complement later life treatment trials. We are only aware of one previous non‐pharmacological intervention trial for the prevention of AD in adults with DS. This study by Sano et al.^48^ examined the use of vitamin E supplementation on cognitive outcomes in 337 individuals with DS and found no difference between the vitamin E and control arms. Thus, non‐pharmacological lifestyle trials in adults with DS are warranted.

Although limited, clinical trials involving adults with overweight and obesity suggest that intentional weight loss is also linked to improvements in cognitive function and a reduced risk of developing AD.^9^, ^10^, ^11^, ^12^ A meta‐analysis^10^ of interventional studies for weight loss identified 13 longitudinal studies (*n* = 551) and seven RCTs (*n* = 468) of participants with overweight (i.e., BMI 25.0–29.9 kg/m^2^) or obesity (i.e., BMI ≥30.0 kg/m^2^). Intentional modest weight loss of even 2 kg among trial participants was associated with improvements in cognition at a 6‐month follow‐up. However, the potential for weight loss and/or changes in dietary intake to prevent or delay AD in adults with DS has not yet been examined.

This randomized trial will be the first study to examine the impact of weight loss on AD biomarkers in adults with DS. Considerable thought went into how to design and implement this trial, and many aspects of the trial were developed with a community research advisory board, consisting of self‐advocates with DS, caregivers, and community agencies servicing people with DS. First, we considered it unethical to randomize adults with DS who represent an underserved group with significant health challenges to a no treatment control condition. We chose general health education as our control arm. This approach will offer something of potential interest and benefit to participants and may also improve participant recruitment. Next, we chose to deliver the intervention remotely. Remote delivery eliminates the need for travel to an intervention site and thus, represents a cost‐effective strategy for the delivery of weight management with the potential for widespread reach, adoption, and implementation. We have previously demonstrated clinically relevant mean weight loss in adults with ID including DS over 24 months that did not differ between in‐person or remote delivery.^18^ Finally, all outcomes proposed for this trial, except for a standard blood draw, are non‐invasive and pose minimal burden and risk to participants. We considered the use of invasive techniques such as positron emission tomography (PET) scans and cerebrospinal fluid; however, we chose to use AD markers obtained from blood samples or MRI as the cost and participant burden associated with these procedures exceeds any potential benefit realized from this initial pilot trial.

Design and methodologic strengths of this trial include (1) randomized design with 2:1 allocation to intervention arms with assignments concealed until the initiation of the intervention; (2) adequate sample size to provide stable estimates of effect sizes per our primary aim; (3) strategies to ensure that we meet our participant recruitment goals; (4) retention/incentive strategies to reduce loss to follow‐up; (5) utilization of cognitive measures, and plasma biomarkers that are being used in the Alzheimer Biomarker Consortium for Down Sydnrome,^30^ the largest US cohort of adults with DS examining biomarkers related to AD, allowing for harmonization of data; (6) the use of a evidenced‐based intervention delivered by trained interventionists; (7) delivery of both intervention arms by the same interventionists to minimize interventionist effects; (8) procedures to ensure intervention fidelity; (9) assessments completed by trained research assistants blinded to intervention arm; (10) evaluation of staff inter‐rater reliability for all physical assessments; (11) the use of a novel technique for the assessment of dietary intake and quality; (12) exit interviews to obtain potentially useful information for improving the intervention; (13) an analysis plan to adequately address study aims. However, the study is not without limitations. Given the exploratory nature of the study not all outcomes will have adequate power for detecting difference between groups, and the 12‐month intervention length may not be long enough to detect changes in AD pathology.

Completion of this project represents an initial step toward developing and disseminating effective interventions to prevent or delay the development of AD in adults with DS. This trial will provide preliminary data on the impact of weight loss and improved diet quality on cognitive function and factors associated with the development of AD and the time course of any changes in adults with DS. Additionally, results of this study could help inform dietary guidelines for adults with DS. If the results of this pilot are positive, that is, improvements in some or all AD associated outcomes assessed we will propose an adequately powered randomized trial to further investigate the impact of weight loss and diet quality on these outcomes in adults with DS. If our pilot results suggest an independent effect of diet quality on factors associated with AD we will also propose an adequately powered randomized trial to investigate the impact of improved diet quality without weight loss on AD outcomes. Subsequent trials may include additional AD markers obtained from cerebrospinal fluid (CSF) or PET scans.

## CONFLICT OF INTEREST STATEMENT

L.T.P. receives funding from the NIH both related and unrelated to this work and research support to conduct clinical trials (paid to institution) from ACI‐24. J.E.D. receives funding from the NIH both related and unrelated to this work. He receives funding for consulting from the University of Colorado and the University of Massachusetts and funding from Health Partners (Minneapolis) for Data Safety and Monitoring Board. J.B. has received research support from the NIH, research support to conduct clinical trials (paid to institution) from Eli Lilly, Amylyx, Biogen, Eisai, AbbVie, Astra‐Zeneca, Roche, and Ionis and has served as a consultant for Renew Research, Eisai, Eli Lilly, LabCorp, Roche, Renew Biotechnologies, AbbVie, Novo Nordisk. J.B. serves on a Data Monitoring Committee for Intra‐Cellular Therapies, Inc. J.M. receives funding from the NIH both related and unrelated to this work, and funding from the University of Kentucky for Data Safety and Monitoring Board. S.H. receives funding from the NIH unrelated to this work. She serves as a consultant to the University of Kansas Medical Center related to this work and has served as a consultant for IONIS Pharmaceuticals. L.M. receives funding from the NIH both related and unrelated to this work. She has received Honoria for a panel discussion for the National Center for Faculty Development and Diversity. M.B. and J.S. receive funding from the NIH both related and unrelated to this work. P.L., I.C., L.K., S.H., J.D., and K.S. receive funding from the NIH related to this work. D.S. receives funding from NIH related and unrelated to this work, as well as funding from HHS, MCHB, U.S Highbush Blueberry Council, and National Cattlemen's Beef Association unrelated to this work. M.B. has nothing to disclose. Author disclosures are available in Supporting Information.

## CONSENT STATEMENT

This article provides the protocol for a clinical trial. All subjects who will enroll in this trial will provide informed consent at the time of enrollment.

## Supporting information



Supporting Information
